# Pathogenic Aβ production by heterozygous PSEN1 mutations is intrinsic to the mutant protein and not mediated by conformational hindrance of wild-type PSEN1

**DOI:** 10.1016/j.jbc.2023.104997

**Published:** 2023-06-30

**Authors:** Vanessa Kurth, Isabella Ogorek, Carolina Münch, Javier Lopez-Rios, Solenne Ousson, Sandra Lehmann, Katja Nieweg, Anton J.M. Roebroek, Claus U. Pietrzik, Dirk Beher, Sascha Weggen

**Affiliations:** 1 Department of Neuropathology, Heinrich Heine University, Düsseldorf, Germany; 2 Institute of Pathobiochemistry, University Medical Center of the Johannes Gutenberg-University, Mainz, Germany; 3 Centro Andaluz de Biología del Desarrollo (CABD), CSIC-Universidad Pablo de Olavide-Junta de Andalucia, Sevilla, Spain; 4 Asceneuron SA, Lausanne, Switzerland; 5 Institute of Pharmacology and Clinical Pharmacy, Philipps-University, Marburg, Germany; 6 Department of Human Genetics, KU Leuven, Leuven, Belgium

**Keywords:** Alzheimer disease, amyloid-beta (Aβ), amyloid precursor protein (APP), presenilin, mutant, gamma-secretase, genome engineering, recombination, embryonic stem cell

## Abstract

Presenilin-1 (PSEN1) is the catalytic subunit of the intramembrane protease γ-secretase and undergoes endoproteolysis during its maturation. Heterozygous mutations in the PSEN1 gene cause early-onset familial Alzheimer’s disease (eFAD) and increase the proportion of longer aggregation-prone amyloid-β peptides (Aβ42 and/or Aβ43). Previous studies had suggested that PSEN1 mutants might act in a dominant-negative fashion by functional impediment of wild-type PSEN1, but the exact mechanism by which PSEN1 mutants promote pathogenic Aβ production remains controversial. Using dual recombinase-mediated cassette exchange (dRMCE), here we generated a panel of isogenic embryonic and neural stem cell lines with heterozygous, endogenous expression of PSEN1 mutations. When catalytically inactive PSEN1 was expressed alongside the wild-type protein, we found the mutant accumulated as a full-length protein, indicating that endoproteolytic cleavage occurred strictly as an intramolecular event. Heterozygous expression of eFAD-causing PSEN1 mutants increased the Aβ42/Aβ40 ratio. In contrast, catalytically inactive PSEN1 mutants were still incorporated into the γ-secretase complex but failed to change the Aβ42/Aβ40 ratio. Finally, interaction and enzyme activity assays demonstrated the binding of mutant PSEN1 to other γ-secretase subunits, but no interaction between mutant and wild-type PSEN1 was observed. These results establish that pathogenic Aβ production is an intrinsic property of PSEN1 mutants and strongly argue against a dominant-negative effect in which PSEN1 mutants would compromise the catalytic activity of wild-type PSEN1 through conformational effects.

The aggregation and accumulation of amyloid-β (Aβ) peptides in the brain is believed to be the initial trigger in the pathogenesis of Alzheimer’s disease (AD), the most common cause of clinical dementia (1). Aβ peptides are generated by sequential proteolysis of the amyloid precursor protein (APP), a ubiquitously expressed type I transmembrane protein. In the Aβ generating pathway, APP is first cleaved in its extracellular juxtamembrane region by β-secretase (β-site APP-cleaving enzyme 1, BACE1) leading to the release of its large ectodomain. The remaining membrane-bound APP C-terminal fragment (APP-CTF, C99) is a substrate for the intramembrane aspartyl protease γ-secretase, which has both endo- and carboxypeptidase activities (2, 3). Endoproteolysis (ε-cleavage) occurs at two adjacent cleavage sites close to the cytosolic end of the C99 transmembrane domain (TMD), resulting in long Aβ peptides of 49 or 48 amino acids (Aβ49, Aβ48) that remain attached to the membrane, and the cytosolic release of the APP intracellular domain (AICD) (4, 5, 6, 7). Subsequently, these long Aβ peptides are cleaved by the carboxypeptidase activity of γ-secretase roughly every three amino acids into shorter Aβ peptides, which are secreted into the extracellular space. Depending on the initial endoproteolytic cleavage site, trimming of the longer peptides proceeds in two major product lines: Aβ49>Aβ46>Aβ43>Aβ40>Aβ37 and Aβ48>Aβ45>Aβ42>Aβ38 (8, 9, 10, 11). In conditioned media of cultured cells, Aβ40 peptides are the predominant species (∼80%) followed by Aβ42 and Aβ38 and minor amounts of Aβ43 and Aβ37 (2). γ-Secretase is a multiprotein complex consisting of the catalytic subunit presenilin (PSEN), either PSEN1 or PSEN2, and three additional membrane proteins, nicastrin (NCT), anterior pharynx-defective 1 (Aph-1), and presenilin enhancer 2 (Pen-2) (3, 12, 13, 14, 15, 16, 17, 18). Aside from APP, over 100 additional γ-secretase substrates have been identified, which have been demonstrated or are assumed to be processed in a similar fashion with endoproteolytic cleavage close to the cytosolic border of the TMD followed by carboxypeptidase trimming and secretion of TMD-derived peptides (3, 19).

Major support for the pivotal role of Aβ in the pathogenesis of AD is drawn from familial cases with early disease onset and autosomal-dominant inheritance (early-onset familial AD, eFAD) (1). The vast majority of patients with eFAD carry heterozygous mutations in the PSEN1 gene, and over 300 mostly missense mutations have been identified that occur throughout the coding region (2, 3, 20, 21, 22). Studies in various model systems including established and eFAD patient-derived cell lines, transgenic mice, human post-mortem tissue, and *in vivo* measurements in patients with eFAD have demonstrated that an increase in the proportion of longer, highly aggregation-prone Aβ peptides (Aβ42, Aβ43) over shorter peptides (Aβ40, Aβ38, Aβ37), typically reported as an increase in the Aβ42/Aβ40 ratio, is likely the common effect of eFAD PSEN1 mutations (23, 24, 25, 26, 27, 28, 29, 30, 31, 32, 33, 34). Strong evidence indicates that this shift in Aβ peptide production by PSEN1 mutations is due to reduced carboxypeptidase processivity in both Aβ product lines. This has been explained by a destabilizing effect of the mutations on the interaction between the enzyme and the Aβ substrate intermediates, resulting in the premature dissociation of longer Aβ peptides from the enzyme (25, 28, 35, 36). In contrast, the initial endoproteolytic cleavage of γ-secretase substrates resulting in the release of intracellular cytoplasmic domains (ICDs) does not seem to be substantially affected by eFAD PSEN1 mutations when examined in eFAD patient-derived brain tissues and cell lines or PSEN1 knock-in mouse models (22, 30, 32, 34, 37, 38, 39, 40). However, for some PSEN1 mutations, subtle changes in the use of the initial endoproteolytic cleavage site might induce more frequent processing of the APP substrate in the Aβ42 generating product line contributing to an increase in the Aβ42/Aβ40 ratio, which is supported by mutagenesis studies indicating changes in the molecular interaction of PSEN1 mutants with the APP substrate (25, 41, 42).

The reduced carboxypeptidase processivity of PSEN1 mutants resulting in a larger proportion of aggregation-prone Aβ42 and Aβ43 peptides has been regarded as a gain-of-function mechanism. Alternatively, the occurrence of PSEN1 missense mutations throughout the coding region without apparent mutational hot spots could be indicative of a loss-of-function mechanism. Furthermore, to explain the autosomal-dominant inheritance pattern associated with heterozygous PSEN1 missense mutations, it has been proposed that PSEN1 mutants are dominant-negative and function as antimorphs *in trans* by interacting with and obstructing the function of the wild-type protein expressed from the second PSEN1 allele (43, 44, 45, 46). Support for this so-called presenilin hypothesis was drawn from several experimental observations including the age-dependent neurodegeneration and memory impairments in conditional PSEN1/PSEN2 double knockout mice (47, 48), the drastically reduced enzyme activity of some PSEN1 mutants when assessed in overexpression or homozygous knock-in models (32, 40, 49, 50, 51), and the large size of γ-secretase complexes observed in early purification attempts consistent with oligomerization of its subunits (52, 53, 54). By transient co-transfection into PSEN-deficient cells, evidence was provided that mutant PSEN1 could perturb the enzymatic activity and Aβ42 generation of wild-type PSEN1 through direct physical interaction, and it was proposed that mutant PSEN1 might allosterically alter the conformation of wild-type PSEN1 (55). Subsequently, it was demonstrated that co-incubation of purified γ-secretase complexes containing wild-type PSEN1 with complexes containing mutant PSEN1 reduced Aβ generation, supporting a dominant-negative effect on enzyme activity through hetero-oligomerization. Moreover, in the presence of different detergents, the proteolytic activity of purified γ-secretase was correlated with its oligomerization (56). In addition, using super-resolution microscopy, a recent study reported that a substantial fraction of γ-secretase in the plasma membrane appeared to be dimeric (57). However, other biochemical and structural studies have provided definitive evidence that the four subunits of the γ-secretase complex are present in a 1:1:1:1 stoichiometry and could not demonstrate an interaction between wild-type and mutant PSEN1 by co-immunoprecipitation (12, 17, 58). These controversial results might in part be related to the predominant use of experimental models that did not accurately resemble the heterozygous genetic background in patients with eFAD in which the mutant PSEN1 allele is expressed in the context of one wild-type PSEN1 and two wild-type PSEN2 alleles. In this study, using genome engineering, we have generated a panel of isogenic murine embryonic stem (ES) cell lines and derived neural stem cells (NSCs) with a heterozygous expression of eFAD PSEN1 mutations. With the ability to discriminate between the two endogenously expressed PSEN1 alleles, we observed no evidence of a physical or functional interaction between wild-type and mutant PSEN1, excluding trans-dominant effects of eFAD PSEN1 mutations.

## Results

**Figure 1:**
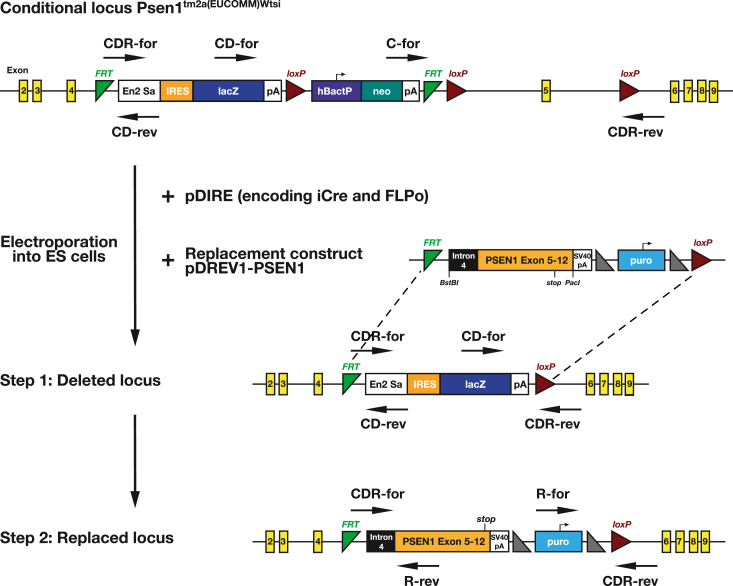
**Dual recombinase-mediated cassette exchange (dRMCE) to introduce heterozygous knock-in mutations into the murine PSEN1 gene.** The dRMCE method takes advantage of conditional alleles generated by the International Knockout Mouse Consortium in which recognition sites for Flpo recombinase (FRT sites), and iCre recombinase (loxP sites) can be exploited to re-engineer the genomic locus with high frequency (59). In the Psen1^tm2a(EUCOMM)Wtsi^ conditional allele, exon 5 is flanked by FRT and loxP sites, which allows to replace the genomic sequence encompassing exons 5 to 12 corresponding to amino acids Ile114 to Ile467. Murine ES cells carrying the PSEN1 conditional allele were co-transfected with a pDREV1-PSEN1 replacement construct and the pDIRE vector. The pDREV1-PSEN1 replacement construct contains a PSEN1 gene fragment consisting of the distal part of intron four and a cDNA fragment of exons 5 to 12, followed by a stop codon and the PSEN1 3′-UTR. This PSEN1 gene fragment is preceded by an FRT site and followed by a puromycin resistance gene and a loxP site. The pDIRE vector encodes both Flpo and iCre recombinases. The recombination between the PSEN1 conditional allele and the replacement construct likely proceeds in two steps. In the first step, with the expression of iCre, the loxP sites in the conditional allele collapse resulting in an intermediate, deleted locus in which the lacZ coding sequence is flanked by one FRT and one loxP site. In the second step, heterologous recombination between the FRT and loxP sites leads to the exchange of the lacZ coding sequence in the deleted locus with the PSEN1 gene fragment present in the pDREV-1-PSEN1 replacement construct (*dashed lines*). The end result is the replaced locus, which is fully functional. Importantly, the inclusion of the distal part of PSEN1 intron four in the replacement construct ensures that mRNA transcribed from the re-engineered genomic locus will be correctly spliced, leading to the fusion of exon four to exons 5 to 12 encoded by the replacement fragment. This sequence of recombination events appears favored, probably due to the higher expression and efficacy of iCre versus Flpo recombinase (59, 86). After selection with puromycin, single-cell colonies were picked and isolated genomic DNA was screened for the desired recombination events by PCR. The horizontal arrows indicate the annealing sites of the validation primers that were used to differentiate between the conditional PSEN1 gene locus, the deleted locus, and the replaced locus (detailed in Table S2). Cell clones with the correct gene replacement were further expanded and cryopreserved.

The majority of patients with eFAD harbor heterozygous missense mutations in the PSEN1 gene. Consequently, faithfully modeling eFAD requires a knock-in strategy to alter just one of the two endogenous PSEN1 gene alleles (21). To introduce heterozygous PSEN1 mutations into murine ES cells, we used dual recombinase-mediated cassette exchange (dRMCE), a gene-targeting strategy first described by Osterwalder *et al.* (59). The dRMCE method takes advantage of pretargeted mouse alleles generated by the International Knockout Mouse Consortium. In these conditional alleles, recognition sites for Flpo recombinase (FRT sites) and iCre recombinase (loxP sites) can be exploited to re-engineer the genomic locus with high frequency to introduce mutations, deletions, tags, or reporter sequences (59). The structure of the Psen1^tm2a(EUCOMM)Wtsi^ conditional allele and our strategy to introduce mutations by dRMCE are illustrated in Figure 1. In the PSEN1 conditional allele, exon five is flanked by FRT and loxP sites. This allows to replace the genomic sequence encompassing exons 5 to 12, which correspond to amino acids Ile114-Ile467 or around 75% of the mouse PSEN1 protein. Murine ES cells carrying the PSEN1 conditional allele were co-transfected with a pDREV1-PSEN1 replacement construct and the pDIRE vector. In the pDREV1-PSEN1 replacement construct, a PSEN1 cDNA fragment of exons 5 to 12 is preceded by an FRT site and followed by a puromycin resistance gene and a loxP site. The pDIRE vector encodes both Flpo and iCre recombinases to facilitate the recombination between the PSEN1 conditional allele and the replacement construct. After selection with puromycin, single-cell colonies were expanded, and isolated genomic DNA was screened for the desired recombination events by PCR. By assessing the transfection of 10 different PSEN1 replacement constructs, we observed that on average 52% of all analyzed single-cell clones displayed the correct gene replacement (Table S3). For each individual replacement construct, it was sufficient to screen just six colonies to obtain at least two positive recombinants. This exceptional efficacy of dRMCE is largely consistent with previously reported findings for four other genomic loci (59).

**Figure 2:**
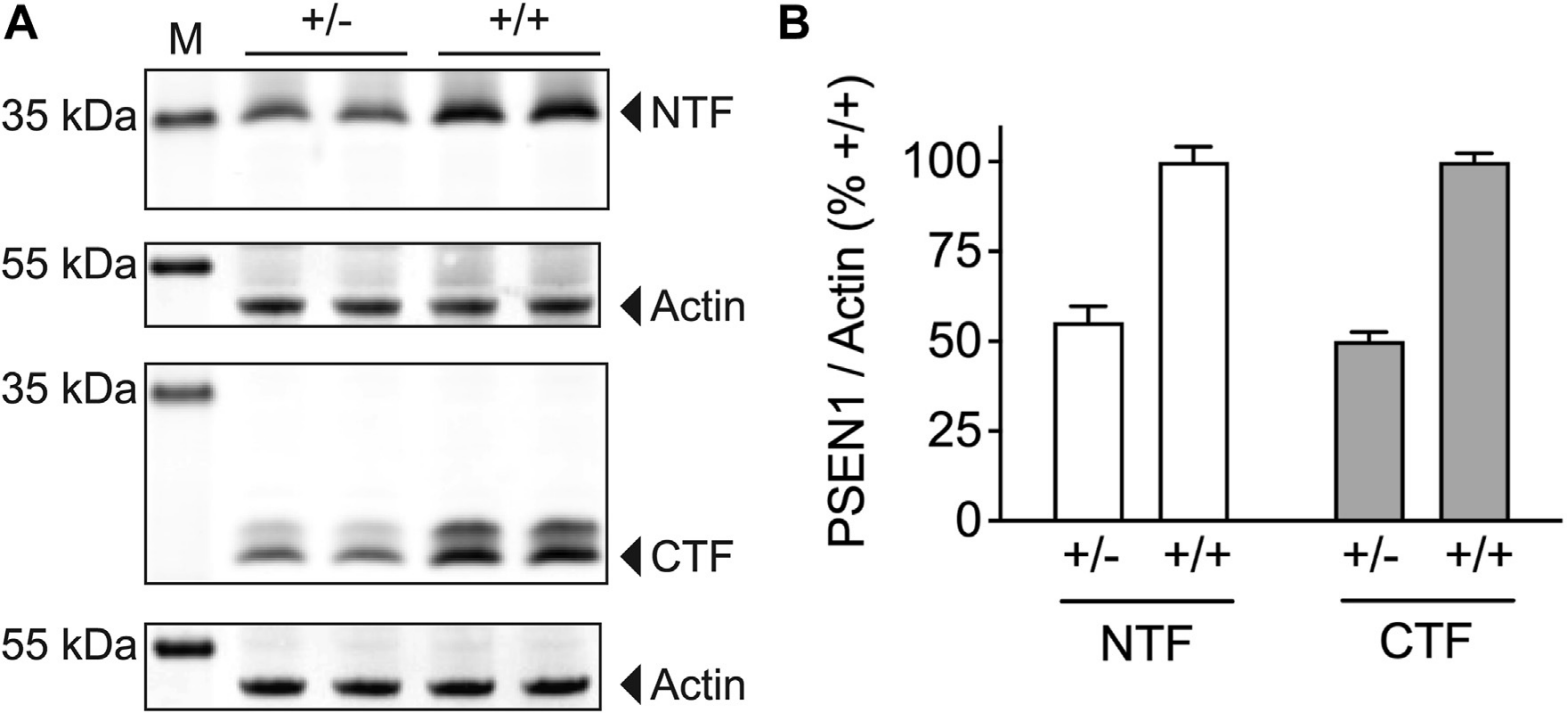
**Successful dRMCE results in a fully functional PSEN1 gene locus.** ES cells carrying the Psen1^tm2a(EUCOMM)Wtsi^ conditional allele were transfected with a replacement construct encoding the wild-type sequence of PSEN1 exons 5 to 12. After drug selection and genotyping of single-cell clones, PSEN1 protein levels were compared between cell clones with successful recombination that displayed the replaced locus (+/+) and cell clones with failed recombination that displayed the deleted locus (+/−). The deleted locus is non-functional and does not lead to the expression of PSEN1. *A*, in cells with the deleted locus (+/−), Western blotting analysis with anti-PSEN1 antibodies detected protein bands with a molecular weight of approximately 35 and 20 kDa representing the N-terminal (NTF) and C-terminal (CTF) fragments of murine PSEN1. The less prominent protein band detected above the PSEN1-CTF likely represents a phosphorylated form of the CTF as shown previously (87). The same protein bands with a higher signal intensity were detected in cells with the replaced locus (+/+). Four independent biological experiments each with two technical replicates were performed (n = 4), and one representative experiment is shown. *B*, quantification of PSEN1-NTF and CTF protein levels. PSEN1-NTF and CTF levels were around twice as high in cells with the replaced locus as compared to cells with the deleted locus, indicating that the replaced locus is functional and expressed in a 1:1 ratio with the second endogenous wild-type PSEN1 allele. The signal intensities of the PSEN1-NTF or CTF were quantified, normalized to actin levels, and averaged from four independent biological experiments (n = 4). The PSEN1 levels of cells with the replaced locus (+/+) were set to 100%, and the levels of cells with the deleted locus (+/−) were calculated as % control ± SD.

For cell clones with the correct gene replacement, it was critical to prove that the re-engineered allele was fully functional and expressed in an equal 1:1 ratio with the second, wild-type PSEN1 allele. To this end, we transfected ES cells carrying the PSEN1 conditional allele with a replacement construct encoding the wild-type sequence of PSEN1 exons 5 to 12. After drug selection and genotyping of single-cell clones, PSEN1 protein levels were compared in cell clones that had undergone successful recombination (replaced locus) with cell clones that had undergone only partial recombination (deleted locus) (Fig. 1). Cell clones with the deleted locus have only one functional PSEN1 allele and would be expected to express only 50% of PSEN1 protein compared to cell clones with the replaced locus. In cells with the deleted locus, Western blotting analysis with anti-PSEN1 antibodies detected protein bands with a molecular weight of approximately 35 and 20 kDa representing the N-terminal (PSEN1-NTF) and C-terminal (PSEN1-CTF) fragments present in the mature γ-secretase complex (Fig. 2*A*). In cells with the replaced locus, the same protein bands with higher signal intensity were observed (Fig. 2*A*), and densitometry analysis demonstrated that PSEN1-NTF and PSEN1-CTF levels were around twice as high in cells with the replaced locus as compared to cells with the deleted locus (Fig. 2*B*). Taken together, dRMCE proved to be a powerful and highly efficient method to introduce heterozygous mutations into the endogenous murine PSEN1 gene locus. Importantly, the re-engineered allele was fully functional and expressed proportionally to the second wild-type PSEN1 allele.

**Figure 3:**
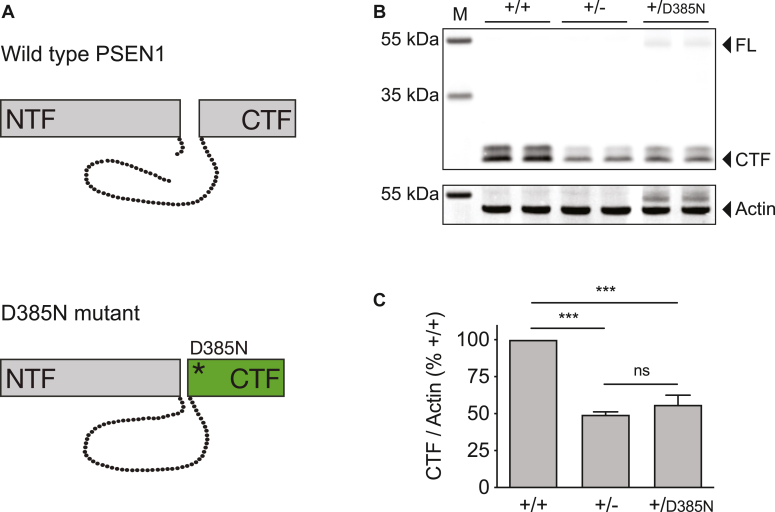
**Endoproteolytic cleavage of PSEN1 does not occur *in trans*.** ES cells carrying the Psen1^tm2a(EUCOMM)Wtsi^ conditional allele were transfected with a replacement construct encoding PSEN1 exons 5 to 12 containing the D385N mutation, and stable recombinants were selected. *A*, in contrast to wild-type PSEN1, the D385N mutant is catalytically-inactive and does not undergo endoproteolysis in its cytoplasmic loop domain during maturation of the γ-secretase complex. *B*, to investigate whether the D385N mutant might be cleaved by the wild-type PSEN1 protein *in trans*, protein levels of the PSEN1 C-terminal fragment (CTF) were compared by Western blotting in ES cells with expression of two wild-type alleles (+/+), in cells with only one functional PSEN1 allele (+/−), and in cells with heterozygous expression of the D385N mutant (+/D385N). The PSEN1-CTF migrated with a molecular weight of approximately 20 kDa with a less prominent band above representing a phosphorylated form of the CTF (87). In +/D385N cells, full-length PSEN1 protein (FL) with a molecular weight of around 55 kDa was also detected. Three independent biological experiments each with two technical replicates were performed (n = 3), and one representative experiment is shown. *C*, quantification showed that PSEN1-CTF levels were twice as high in ES cells with two wild-type PSEN1 alleles compared to cells with only one functional PSEN1 allele or cells with heterozygous expression of the D385N mutant. No statistically significant difference in PSEN1-CTF levels was observed between +/− and +/D385N cells indicating that PSEN1 endoproteolysis is strictly an intramolecular event. The signal intensities of the PSEN1-CTF were quantified, normalized to actin levels, and averaged from three independent biological experiments (n = 3). The PSEN1-CTF levels of +/+ cells were set to 100%, and the levels of +/− and +/D385N cells were calculated as % control ± SD. The mean relative expression levels of the cell lines were compared by one-way ANOVA with Tukey’s post tests. ∗∗∗*p* < 0.001; ns, not significant.

During assembly and maturation of the γ-secretase complex, PSEN1 undergoes endoproteolysis in its large cytoplasmic loop domain linking transmembrane domains six and 7 with predominant cleavage between Met298 and Ala299 (60, 61). The resulting PSEN1-NTF and CTF remain non-covalently attached, and the endoproteolysis step is believed to coincide with the final activation of the enzyme complex and has been demonstrated to occur by autoproteolysis. Mutation of either of the catalytic aspartate residues Asp257 and Asp385 blocks endoproteolysis of PSEN1, and the stepwise cleavage mechanism of PSEN1 endoproteolysis is highly reminiscent of the proteolytic processing of γ-secretase substrates (62, 63). To examine if two or more PSEN1 molecules might functionally interact in a cellular model with physiological allele expression, we first asked whether endoproteolysis of PSEN1 occurred strictly as an intramolecular event or might also result from the intermolecular interaction of two PSEN1 molecules *in trans*. To investigate this issue, we replaced the PSEN1 conditional allele in ES cells with a cDNA fragment of exons 5 to 12 containing the D385N mutation. This mutation targets one of the two critical aspartate residues in PSEN1 and results in a mutant that is catalytically inactive, does not undergo endoproteolysis, and accumulates as a full-length protein (62, 63) (Fig. 3*A*). We then compared the protein levels of the PSEN1-CTF in ES cells with the expression of two wild-type alleles (+/+), in ES cells that had failed to undergo successful recombination with only one functional PSEN1 allele (+/−), and in ES cells in which the catalytically-dead D385N mutant was expressed alongside the wild-type PSEN1 protein (+/D385N) (Fig. 3*B*). Quantification of Western blots confirmed that PSEN1-CTF levels were twice as high in ES cells with two wild-type PSEN1 alleles compared to ES cells with only one functional PSEN1 allele (Fig. 3*C*). ES cells with heterozygous expression of the D385N mutant displayed approximately half the amount of PSEN1-CTF compared to cells with two wild-type alleles, with no statistically significant difference compared to the PSEN1-CTF levels in ES cells with only one functional PSEN1 allele. In addition, full-length PSEN1 was present in ES cells with heterozygous expression of the catalytically-dead D385N mutant (Fig. 3*B*). In sum, these findings provided evidence that the D385N mutant is not cleaved *in trans* by the wild-type protein and indicated that PSEN1 endoproteolysis exclusively occurred as an intramolecular event.

**Figure 4:**
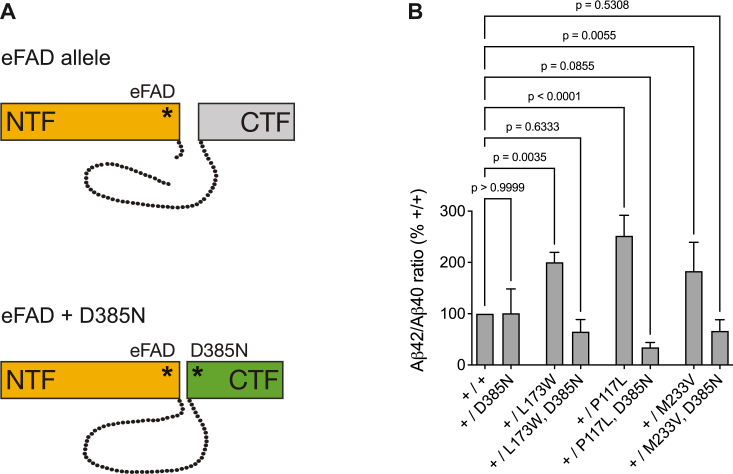
**eFAD-associated PSEN1 mutants require catalytic activity to induce a pathogenic increase in the Aβ42/Aβ40 ratio.***A*, by dRMCE, ES cell lines with a heterozygous expression of the eFAD-associated PSEN1 mutations P117L, L173W, and M233V were generated. In additional ES cell lines, each of these eFAD mutations was combined with the D385N mutation targeting a critical active site aspartate residue. These PSEN1 double-mutants are catalytically inactive and not cleaved into NTF and CTF fragments during the maturation of the γ-secretase complex. *B*, the ES cell lines were differentiated into neural stem cells (NSCs) and infected with an adenovirus encoding the human APP695 isoform with the Swedish mutation. In conditioned tissue culture media, secreted Aβ40 and Aβ42 levels were quantified by an electrochemical luminescence-based immunoassay. NSC lines with heterozygous expression of the eFAD PSEN1 mutations displayed a significantly increased Aβ42/Aβ40 ratio as compared to NSCs with two wild-type PSEN1 alleles (+/+) or with only one functional PSEN1 allele (+/D385N). In contrast, NSC lines expressing the catalytically-dead eFAD mutants showed a normal Aβ42/Aβ40 ratio not different from NSCs with two wild-type PSEN1 alleles. The mean Aβ42/Aβ40 ratios of the NSC lines were calculated from three to five independent biological experiments (n = 3–5). The ratio of NSCs with two wild-type PSEN1 alleles (+/+) was set to 100%, and the ratios of all other NSC lines were calculated as % control ± SD. The Aβ42/Aβ40 ratios of all NSC lines were compared to +/+ cells by one-way ANOVA with Tukey’s post tests.

A number of studies have reported potential trans-dominant effects of eFAD-associated PSEN1 mutants on wild-type PSEN1 in a dimeric or oligomeric complex (32, 44, 55, 56). To address this controversial issue in a cellular model with heterozygous and endogenous expression of PSEN1 mutations, three mutations were chosen that cause eFAD in the second or third decade of life (64, 65, 66), and were shown to promote a substantial increase in secreted Aβ42 levels and the Aβ42/Aβ40 ratio compared to wild-type PSEN1 after stable overexpression in HEK293 cells (67). Using dRMCE, ES cell lines with a heterozygous expression of either the PSEN1 P117L, the L173W, or the M233V mutations were generated (Fig. 4*A*). In a second set of ES cell lines, each of these eFAD mutations was combined with the D385N mutation targeting one of the two catalytic aspartate residues. These PSEN1 double-mutants are catalytically inactive and not cleaved into NTF and CTF fragments due to the lack of autoproteolysis and *trans* cleavage by wild-type PSEN1 ((62, 63), and Fig. 3). This ensured that a PSEN1 NTF containing the eFAD mutation would not be able to assemble with a CTF expressed from the wild-type PSEN1 allele to form a mutant but catalytically competent γ-secretase enzyme complex (Fig. 4*A*). In this experimental system, cell lines with a heterozygous expression of the eFAD mutations would be expected to display an increase in the Aβ42/Aβ40 ratio compared to control cells harboring two wild-type PSEN1 alleles. However, any change in the Aβ42/Aβ40 ratio in cell lines expressing the PSEN1 double-mutants would provide strong evidence for a functional interaction between mutant and wild-type PSEN, and indicate that the mutant protein can influence the processing of substrate by the wild-type protein, presumably by a trans-dominant conformational effect. To study the mutational effects in a neuronal cell type, the ES cell lines were differentiated into NSCs, which displayed typical morphology, expression of the stem cell markers SOX2 and Nestin and strongly upregulated protein expression of the β-secretase BACE1 compared to the parental ES cells (Fig. S1). All NSC lines expressed comparable levels of the γ-secretase subunits NCT and PEN-2 (Fig. S2). As expected, the cell lines with only one catalytically active PSEN1 allele displayed lower PSEN1-CTF levels accompanied by full-length PSEN1. Importantly, these NSC lines did not show compensatory upregulation of PSEN2 protein levels (Fig. S2). To enable Aβ measurements, the NSC lines were infected with an adenovirus encoding human APP695, and secreted Aβ40 and Aβ42 levels were quantified in conditioned tissue culture media by an electrochemical luminescence-based immunoassay (Table S4). All NSC lines with heterozygous expression of an eFAD PSEN1 mutation displayed a significantly increased Aβ42/Aβ40 ratio as compared to NSCs with two wild-type PSEN1 alleles (+/+) or with only one functional PSEN1 allele (+/D385N) (Fig. 4*B*). In contrast, all NSC lines expressing a catalytically-dead eFAD mutant showed a normal Aβ42/Aβ40 ratio not different from NSCs with two wild-type PSEN1 alleles. These results established that heterozygously expressed eFAD mutants require catalytic activity to induce a pathogenic change in the Aβ42/Aβ40 ratio, and they provided no evidence for trans-dominant effects of eFAD mutants and functional interaction between the two PSEN1 alleles.

**Figure 5:**
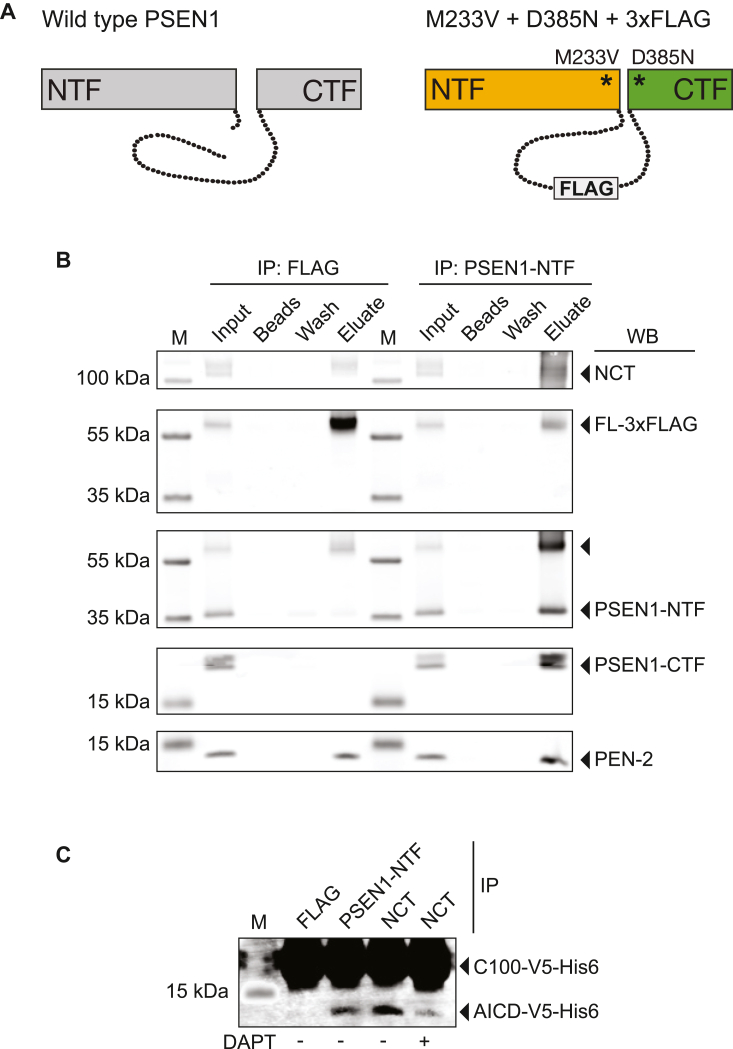
**Co-immunoprecipitation and γ-secretase *in vitro* assays provide no evidence for the interaction of wild-type and eFAD mutant PSEN1.***A*, to probe a putative physical interaction between wild-type and eFAD mutant PSEN1, an ES cell line was created in which the wild-type PSEN1 allele was expressed alongside a mutant allele with the M233V eFAD mutation, the D385N mutation targeting the catalytic aspartate, and a 3xFLAG tag inserted into the cytosolic loop domain. The 3xFLAG tag and the lack of endoproteolytic cleavage allowed to discriminate between the mutant and the wild type PSEN1 proteins by immunoprecipitation and Western blotting. *B*, CHAPSO-solubilized membranes were prepared and immunoprecipitations were performed with an antibody against the PSEN1-NTF or with anti-FLAG antibody. The immunoprecipitated material (eluate) was analyzed by Western blotting with antibodies against the different γ-secretase subunits. The PSEN1-NTF antibody immunoprecipitated both the NTF expressed from the wild-type PSEN1 allele and the eFAD mutant full-length protein (FL-3xFLAG), which migrated with a molecular weight slightly above 55 kDa. Co-immunoprecipitation of the γ-secretase subunits Nicastrin (NCT) and presenilin enhancer 2 (PEN-2) and of the PSEN1-CTF expressed from the wild-type PSEN1 allele was detected. Co-immunoprecipitation of NCT and PEN-2 was also observed when the anti-FLAG antibody was used for immunoprecipitation of the eFAD mutant full-length protein. In contrast, co-immunoprecipitation of the PSEN1-NTF or the PSEN1-CTF expressed from the wild-type allele was not detected. Control immunoprecipitations were performed in which detergent-solubilized membranes were incubated with agarose beads but without the primary antibody (beads). In all experiments, non-specific binding of minimal amounts of the PSEN1-NTF to the bead material was observed as previously reported (17). In the input lane, one-eighth of the total protein amount used for the immunoprecipitations was loaded. In the wash lane, one-fortieth of the total wash fraction (5 μl of 200 μl) was loaded. Three independent biological experiments were performed (n = 3), and one representative experiment is shown. *C*, for γ-secretase *in vitro* assays, CHAPSO-solubilized membrane preparations were immunoprecipitated with antibodies against NCT, PSEN1-NTF, or with anti-FLAG antibody, and the immunoprecipitated material was incubated with a recombinant V5-tagged APP-C100 substrate. Immunoprecipitation with antibodies against NCT and PSEN1-NTF led to the formation of the APP intracellular domain (AICD), which was detected by Western blotting with anti-V5 antibody. Co-incubation of the reaction mixture with the γ-secretase inhibitor DAPT resulted in reduced AICD formation. Conversely, when the anti-FLAG antibody was used for immunoprecipitation of the eFAD mutant PSEN1 protein, no AICD was detected, indicating that the mutant protein did not co-precipitate active γ-secretase complexes containing wild/type PSEN1 protein. Two independent biological experiments were performed (n = 2), and one representative experiment is shown.

To investigate whether any physical interaction is detectable between wild-type and eFAD mutant PSEN1, we used dRMCE to create an ES cell line in which the wild-type PSEN1 allele was expressed alongside a mutant allele with the M233V eFAD mutation, the D385N mutation targeting the catalytic aspartate, and a 3xFLAG tag inserted into the large cytosolic loop domain of PSEN1 (Fig. 5*A*). In this position, a much larger GFP tag had previously been shown not to interfere with the catalytic activity of the γ-secretase complex (68). The 3xFLAG tag in conjunction with the lack of endoproteolytic cleavage enabled unambiguous discrimination between the mutant and the wild-type PSEN1 proteins in this ES cell line by immunoprecipitation and Western blotting. Detergent-solubilized membranes were prepared from the ES cells in 1% CHAPSO, and immunoprecipitations were performed either with an antibody against the PSEN1-NTF or with anti-FLAG antibody. The PSEN1-NTF antibody was able to pull down both the NTF expressed from the wild-type PSEN1 allele and the full-length protein expressed from the mutant allele. Accordingly, co-immunoprecipitation of the γ-secretase subunits NCT and PEN-2, and the PSEN1-CTF expressed from the wild-type PSEN1 allele was observed (Fig. 5*B*). When the anti-FLAG antibody was used to pull down the eFAD mutant full-length protein, co-immunoprecipitation of NCT and PEN-2 was also seen, which confirmed that the catalytically inactive mutant protein was normally incorporated into the γ-secretase complex, consistent with previous results (17, 69). However, co-immunoprecipitation of the PSEN1-NTF or the PSEN1-CTF expressed from the wild-type allele was not detected, providing no evidence for interaction between the eFAD mutant and the wild-type PSEN1 proteins (Fig. 5*B*). In all experiments, minimal amounts of the PSEN1-NTF were observed in the eluate fraction. This was also seen in the wash fraction and after the incubation of detergent-solubilized membranes with agarose beads but without the anti-FLAG antibody, clearly demonstrating that a small amount of the hydrophobic PSEN1-NTF binds non-specifically to the bead material as previously reported (17). Comparable results were obtained with a similarly constructed ES cell line in which the mutant allele incorporated the L166P eFAD mutation (Fig. S3). This mutation is also associated with a very early onset of clinical symptoms in the second decade of life, and it has been proposed to exert dominant-negative effects through hetero-oligomerization of wild-type PSEN1 (56, 70). In addition, we performed γ-secretase *in vitro* assays to probe whether immunoprecipitation of the catalytically-dead eFAD mutant might co-precipitate any active γ-secretase (Fig. 5*C*). As a positive control, an antibody against NCT was employed and the immunoprecipitated material was incubated with a recombinant V5-tagged APP-C100 substrate. This resulted in the generation of the APP intracellular domain (AICD), which was diminished by co-incubation of the reaction mixture with the γ-secretase inhibitor DAPT. Likewise, when an antibody against the PSEN1-NTF was used, which was able to immunoprecipitate both the wild-type protein and the catalytically dead PSEN1 mutant, AICD formation was observed. In contrast, when the anti-FLAG antibody was used for immunoprecipitation, no AICD was generated indicating that the mutant protein did not bind and co-precipitate active γ-secretase complexes containing wild-type PSEN1 protein (Fig. 5*C*).

## Discussion

Previous studies of PSEN1 mutations have mostly been conducted in model systems with either transient of stable overexpression. While high-level overexpression is a liability for the investigation of any protein (71), it appears particularly problematic to study the pathogenic effects of PSEN1 mutants (21). As the formation of mature and enzymatically active γ-secretase complexes within a cell is restricted by the limited supply of γ-secretase subunits, the overexpression of PSEN1 mutants in established cell lines results in the replacement of endogenous PSEN1 and PSEN2 proteins from the enzyme complexes (72). Alternatively, PSEN1 mutants have been overexpressed in PSEN1/PSEN2-deficient cells (49). In both of these tissue culture models, functional γ-secretase complexes incorporate mostly or exclusively the exogenously expressed PSEN1 mutants. This is fundamentally different from the physiological condition in patients with eFAD carrying heterozygous PSEN1 mutations, who express one wild/type and one mutant PSEN1 allele and two wild-type PSEN2 alleles (21). Why the equal co-expression of mutant and wild-type PSEN1 alleles is critical is exemplified by our evolved understanding of to what degree PSEN1 mutants affect the initial endoproteolytic cleavage and the release of ICDs from γ-secretase substrates. Earlier studies using transient or stable overexpression of PSEN1 mutants had reported accumulation of γ-secretase substrates and reduced formation of ICDs (21, 49, 51, 70, 73, 74). In contrast, more recent studies using eFAD patient-derived cell lines, human brain tissues from patients with eFAD, and PSEN1 knock-in mouse models have found no or only minor reductions of the endoproteolytic cleavage activity, indicating that in the heterozygous context, the co-expressed wild-type allele compensates for the reduced proteolytic activity of PSEN1 mutants (22, 30, 32, 34, 37, 38, 39, 40). In this study, we have used genome engineering by dRMCE to introduce heterozygous mutations and tags into the murine PSEN1 gene. The dRMCE method allows the introduction of complex modifications to a gene of interest and is based on conditional alleles in murine ES cells that have been generated for thousands of genes by the International Knockout Mouse Consortium (59). Consistent with previous results for different gene loci (59), we found dRMCE to be highly efficient with around 50% of all replacement events resulting in a correctly modified PSEN1 gene locus. Although restricted to murine ES cells, this striking efficacy of dRMCE offers a distinct advantage over CRISPR/Cas9-mediated genome engineering, which for heterozygous gene modifications remains challenging and laborious even with optimized protocols (75).

We have used our dRMCE-generated panel of isogenic ES cell lines to reconsider the controversial issues of PSEN1 oligomerization and dominant-negative effects of eFAD PSEN1 mutations. First, we examined whether endoproteolysis of PSEN1 could result from the intermolecular interaction of two PSEN1 molecules *in trans*. An ES cell line with the heterozygous expression of the D385N mutation targeting one of the two active site aspartates was created, which resulted in a catalytically-inactive mutant that does not undergo autoproteolysis but retains a wild-type endoproteolytic cleavage site (62, 63). In this experimental setting, the D385N mutant was not cleaved *in trans* by the catalytically-competent wild-type PSEN1 protein. The conclusion that PSEN1 endoproteolysis is strictly an intramolecular event is fully consistent with earlier findings by Brunkan *et al.* (76). These authors had transiently co-expressed wild-type PSEN1 incorporating a large N-terminal tag alongside the catalytically-inactive PSEN1 D257A aspartate mutant in PSEN1/PSEN2-deficient cells. Under these conditions, a smaller PSEN1-NTF with faster migration behavior was not detected by Western blotting, indicating that the D257A mutant was not cleaved by the tagged wild-type protein. However, notably, the authors did claim a direct interaction between the wild-type and the mutant proteins in co-immunoprecipitation experiments, a finding that we were unable to confirm as discussed below (76).

In ES cell-derived NSCs, we further explored whether a pathogenic increase in the Aβ42/Aβ40 ratio requires catalytic activity or might also occur through trans-dominant conformational effects of eFAD PSEN1 mutants. Our experimental design was in part adapted from a study by Schroeter *et al.* in which a non-significant trend for an elevated Aβ42/Aβ40 ratio was observed after transient co-expression of wild-type PSEN1 with a catalytically-dead eFAD mutant (M146L/D385A double-mutation) in PSEN1/PSEN2-deficient cells (44). Subsequently, Heilig *et al.* proposed that eFAD PSEN1 mutants act in a trans-dominant fashion to suppress Aβ40 and to stimulate Aβ42 production by wild-type PSEN1, thereby provoking an elevated Aβ42/Aβ40 ratio (55). This was based on the transient co-expression of wild-type PSEN1 with catalytically competent eFAD mutants in PSEN1/PSEN2-deficient cells, which resulted in Aβ40 and Aβ42 levels lower and higher than expected from simply adding the values obtained after single expression of either wild-type or mutant PSEN1. In the same experimental setup, co-immunoprecipitation studies supported the binding of wild-type to mutant PSEN1 although the interaction occurred mainly between full-length PSEN1 proteins indicating that it might have taken place early in the secretory pathway and prior to the formation of the mature γ-secretase complex (55). Importantly, these results were in stark contrast to earlier studies by Sato *et al.*, which, using a very similar experimental design, could not find evidence for a functional or physical interaction between wild-type and mutant PSEN1 (17). A major difference between the two studies was the choice of detergent to solubilize γ-secretase, with Heilig *et al.* reporting the use of CHAPSO (55), while Sato *et al.* employed digitonin to minimize nonspecific interactions of PSEN1 that occurred in their study with CHAPSO (17). Activity of γ-secretase in digitonin is low but Sato *et al.* demonstrated that immunoprecipitation of the γ-secretase complex from digitonin-solubilized membrane preparations and resuspension into CHAPSO resulted in recovery of enzyme activity, proving that essential protein interactions were preserved in digitonin (17). Most recently, Zhou *et al.* reported that Aβ generation by purified γ-secretase containing wild-type PSEN1 was suppressed after mixing with an excess of γ-secretase containing eFAD mutant PSEN1. These authors also claimed that the choice of detergent for γ-secretase solubilization was critical, and they observed an interaction of wild-type and mutant PSEN1 and evidence of γ-secretase oligomerization in co-immunoprecipitation, analytical ultracentrifugation, and electron microscopy studies after solubilization in CHAPSO but not in digitonin (56). Our experiments were designed to avoid the potential drawbacks of these earlier studies. Instead of a cell-free system or ectopic overexpression, we used a cellular system with heterozygous, endogenous expression of eFAD PSEN1 mutations to avoid an overrepresentation of mutant PSEN1 in our activity and binding assays. We further used CHAPSO for solubilization as this detergent has been universally recognized to preserve the integrity and enzymatic activity of the γ-secretase complex. With this experimental setup, we demonstrated for particularly aggressive eFAD PSEN1 mutations that a pathogenic increase in the Aβ42/Aβ40 ratio is strictly dependent on the enzymatic activity of the mutant protein and not mediated by a trans-dominant conformational effect on wild-type PSEN1. These results are consistent with previous findings for the PSEN1-L435F mutation, an eFAD mutation with very low carboxypeptidase activity that predominantly generates Aβ43 peptides. For this mutation, it was demonstrated that Aβ43 generation was attributable to the residual catalytic activity of the mutant protein and did not require co-expression of wild-type PSEN1 (29). In full agreement with the findings by Sato *et al.* (17), our co-immunoprecipitation studies and γ-secretase activity assays failed to provide any evidence for a physical interaction between wild-type and mutant PSEN1. While specific pull-down of mutant PSEN1 confirmed binding to the γ-secretase subunits NCT and PEN2, co-precipitation of the NTF and CTF expressed from the wild-type PSEN1 allele or of any γ-secretase enzyme activity was not detected. A limitation of our study is that combining eFAD mutations with a mutation targeting one of the catalytic aspartates might result in an aberrant protein conformation that renders PSEN1 or the γ-secretase complex unable to oligomerize. However, because of several previous observations, we regard this as unlikely. First, in the naturally occurring eFAD mutation ΔExon9 the endoproteolytic cleavage site is deleted but the protein is functional within the γ-secretase complex indicating that endoproteolysis is not obligatory for PSEN1 to acquire a functional conformation (77). Second, others and we have demonstrated that PSEN1 aspartate mutants are normally incorporated into the γ-secretase complex (17, 69). And third, a recent structure of γ-secretase in complex with an APP substrate, which made use of the PSEN1 D385A catalytic mutant to prevent substrate cleavage, displayed some conformational changes in PSEN1 to accommodate the substrate but the overall structure of γ-secretase and of PSEN1 within the enzyme complex was very similar to an earlier structure of catalytically-competent γ-secretase (12, 78).

In conclusion, our experimental system, which closely resembled the expression of PSEN1 mutations in eFAD patients, revealed that pathogenic Aβ production is an intrinsic property of the mutant protein, providing no support for a dominant-negative effect through oligomerization of wild-type PSEN1.

## Experimental procedures

### Antibodies

The following primary antibodies were used in this study: anti-PSEN1-CTF (rabbit mAb clone D39D1, immunoblotting: 1:1,000, Cell Signaling Technology Cat. No. 5643); anti-PSEN1-NTF (rabbit pAb, immunoblotting: 1:200, immunoprecipitation: 1 μg per 40 μg of solubilized membrane proteins, Santa Cruz Biotechnology Cat. No. sc-7860); anti-Nicastrin (rabbit pAb, immunoblotting: 1:1,000, immunoprecipitation: 1 μg per 40 μg of solubilized membrane proteins, Merck Cat. No. N1660); anti-PEN2 (rabbit pAb, immunoblotting: 1:1,000, Cell Signaling Technology Cat. No. 5451); anti-PSEN2 (rabbit mAb clone D30G3, immunoblotting: 1:1,000, Cell Signaling Technology Cat. No. 9979); anti-BACE1 (rabbit mAb clone D10E5, immunoblotting: 1:1,000, Cell Signaling Technology Cat. No. 5606); anti-SOX2 (rat mAb clone Btjce, immunocytochemistry: 1:100, ThermoFisher Scientific Cat. No. 14-9811-82); anti-Nestin (mouse mAb clone rat-401, immunocytochemistry: 1:100, Developmental Studies Hybridoma Bank); anti-Aβ17-24 (mouse mAb clone 4G8, BioLegend Cat. No. 800710); anti-Aβ40 (mouse mAb clone G2-10, Merck Cat. No. MABN11); anti-Aβ42 (mouse mAb clone A387, licensed from Torrey Pines Therapeutics); anti-Actin (rabbit pAb, immunoblotting: 1:10,000, Merck Cat. No. A2066); anti-V5 Tag (mouse mAb, immunoblotting: 1:500, ThermoFisher Scientific Cat. No. R960-25); anti-FLAG Tag (mouse mAb clone M2, immunoblotting: 1:1,000, immunoprecipitation: 1 μg per 40 μg of solubilized membrane proteins, Merck Cat. No. F3165).

### Cultivation of murine embryonic stem cells

A murine embryonic stem (ES) cell line with a PSEN1 conditional allele (Mouse Genome Informatics ID: Psen1^tm2a(EUCOMM)Wtsi^) based on the JM8A3.N1 parental line (79) and generated by the International Knockout Mouse Consortium (https://www.mousephenotype.org/) was purchased from the European Mouse Mutant Cell Repository (EuMMCR) (Clone ID: EPD0794_4_A06). ES cells were cultured in complete Knockout DMEM containing 10% FBS (v/v), 2 mM L-glutamine, 1× penicillin-streptomycin, 0.1 mM β-mercaptoethanol and 1 × 10^3^ U/ml LIF on gelatin-coated cell culture plates (0.1% gelatin (v/v) in Dulbecco’s PBS without calcium/magnesium (PBS−/−). LIF and gelatin solution from Merck, all other reagents from ThermoFisher Scientific. Medium was exchanged daily and subconfluent ES cells were passaged every 2 days. Detailed protocols for the cultivation of JM8 and other ES cell lines are available on the EuMMCR website (https://www.eummcr.org/protocols/tissue-culture) and in textbooks (80).

### Construction of PSEN1 replacement vectors

To generate the conditional allele Psen1^tm2a(EUCOMM)Wtsi^, a synthetic sequence containing a lacZ reporter gene flanked by FRT and loxP sites was introduced into the intron sequence between exons four and five of the murine PSEN1 locus, with an additional loxP site targeted to the intron sequence between exons five and 6 (Fig. 1). To reconstitute a functional PSEN1 gene locus, a replacement vector was constructed in two steps. First, a synthetic DNA fragment consisting of the distal part of the intron sequence between exons four and 5 (445 bp) of the murine PSEN1 gene, followed by a cDNA fragment of exons 5 to 12 including the stop codon, followed by the PSEN1 3′-UTR, and flanked by 5′-BstBI and 3′-PacI restriction sites was generated by gene synthesis and inserted into the pUC57 plasmid vector (GeneScript). In the second step, this BstBI-PacI fragment with a total size of 2653 bp was subcloned into the pDREV1 vector (a gift from Rolf Zeller, Addgene plasmid # 26751) resulting in the pDREV1-PSEN1 replacement construct. In the replacement construct, the PSEN1 gene fragment is preceded by an FRT site and followed by a puromycin resistance gene and a loxP site (Fig. 1). To introduce additional point mutations into PSEN1 exons 5 to 12, the pDREV1-PSEN1 vector was subjected to single or multiple rounds of mutagenesis using the QuikChange II Site-Directed Mutagenesis Kit (Agilent) according to the manufacturer’s instructions. A 3xFlag tag was introduced into the large hydrophilic loop domain of PSEN1 between amino acids H351 and R352 by overlap extension PCR. Mutagenesis primers were designed using the QuikChange Primer Design Program (Agilent) or manually, and primer sequences are listed in Table S1. All replacement vectors were verified by DNA sequencing.

### Dual recombinase-mediated cassette exchange and PCR validation of recombination events in single-cell clones

The dRMCE gene-targeting method takes advantage of conditional mouse alleles in which recognition sites for Flpo recombinase (FRT sites) and iCre recombinase (loxP sites) can be exploited to re-engineer a genomic locus with high frequency. This method was invented by Osterwalder *et al.* and we performed it as described by the authors with minor modifications (59). To introduce heterozygous mutations into the murine PSEN1 gene locus, an ES cell line with a PSEN1 conditional allele was co-transfected with a pDREV1-PSEN1 replacement construct and the pDIRE vector, which encodes both Flpo recombinase under control of the phosphoglycerate kinase 1 (PGK) promoter and iCre under the eukaryotic translation elongation factor 1 α (EF-1α) promoter (a gift from Rolf Zeller, Addgene plasmid # 26745). 1.5 × 10^7^ cells of the Psen1^tm2a(EUCOMM)Wtsi^ ES cell line were resuspended in 800 μl of PBS−/−, 50 μg each of the pDREV1-PSEN1 and the pDIRE vectors were added, and the cells were electroporated in a 0.4 cm cuvette using a Gene Pulser Xcell Eukaryotic Electroporation System (BIO-RAD) with the following parameters: 0.24 kV, 475 μF, one pulse ∼ 8 ms. After the electroporation, the cuvette was incubated for 20 min on ice, and one-fifth of the cell suspension was then added to 10 ml complete Knockout DMEM containing LIF and plated on a gelatin-coated 10 cm culture dish. Medium was exchanged daily, and 48 h after the electroporation 0.75 mg/ml puromycin was added to select stable cell clones. Figure 1 illustrates the likely sequence of recombination events that take place after the expression of the recombinases as determined by Osterwalder *et al.* (59). First, recombination between the loxP sites in the conditional allele leads to an intermediate, deleted locus. In the second step, FRT and loxP sites in the deleted locus recombine with the same sites in the pDREV-1-PSEN1 replacement construct resulting in the replaced locus, which is fully functional. Approximately 10 to 12 days after the electroporation, single-cell colonies with a round, three-dimensional, and dense morphology were manually isolated with a pipette. Based on preliminary efficacy estimates of the dRMCE procedure, six colonies for each PSEN1 replacement construct were expanded for further validation. Genomic DNA was extracted with the PureLink Genomic DNA Mini Kit (ThermoFisher Scientific), and screening for the desired recombination events was performed by PCR with primer pairs specific for the original conditional PSEN1 gene locus, the deleted locus, and the replaced locus. All positive cell clones were further screened for the potential genomic integration of the Flpo or iCre coding sequences by PCR, which was not observed in any of the investigated clones. The annealing sites for the validation primers are illustrated in Figure 1, and the specificity of the primer pairs for the different loci, the size of the resulting PCR products, and the primer sequences are provided in Table S2. Cell clones with the correct gene replacement were further expanded and cryopreserved.

### Differentiation of ES cells into NSCs

ES cell lines were differentiated into NSCs using general procedures described by Conti *et al.* (81). 6-well plates were coated with Matrigel solution (42 μg/ml in DMEM/F-12, HEPES) and 1.25 × 10^5^ ES cells were plated per well in complete Knockout DMEM containing LIF. After 24 h, the cells were switched to complete DMEM/F-12 medium (DMEM/F-12, HEPES containing 1× penicillin-streptomycin, 0.5× B-27 supplement, 0.5× N-2 supplement, and 0.1 mM β-mercaptoethanol. Matrigel from BD Biosciences, all other reagents from ThermoFisher Scientific). After 5 to 10 days in culture with a daily medium exchange, the ES cells had differentiated into morphologically distinct neuroepithelial precursor cells (NEPs) forming rosette structures. These NEPs were detached from the plate, dissociated using Accutase solution (Merck), resuspended in 5 ml of NSC medium (DMEM/F-12, HEPES containing 1 mM L-glutamine, 1× penicillin-streptomycin, 0.5× B-27 supplement, 0.5× N-2 supplement, and 5 μg/ml heparin (Merck)) containing 10 ng/ml each of EGF and FGF-2 (both from PeproTech), and transferred to an uncoated T25 tissue culture flask. Every 2 days, 1 ml of NSC medium containing EGF and FGF-2 (final concentration of 10 ng/ml each in the total volume of medium) was added. Approximately 5 days after transfer of the NEPs, a sufficient number of floating neurospheres had formed, which were collected, dissociated with Accutase, and replated on laminin (4 ug/ml in PBS−/−, Merck) coated 6-well plates. Subsequently, the cells attached to the laminin surface, migrated out, and formed a monolayer of NSCs, which were propagated in NSC medium containing EGF and FGF-2 with daily medium exchange. NSCs derived by this protocol expressed the self-renewal marker Sox2 and the intermediate filament Nestin and displayed strong upregulation of BACE1 protein expression compared to the parental ES cells (Fig. S1).

### Adenovirus infection of NSCs and immunoassay detection of secreted Aβ peptides

Construction, packaging, and titering of an adenoviral vector encoding the human APP695 isoform with the Swedish mutation have been previously described, and the functional titer of the adenovirus stock was determined to be 5 × 10^8^ transduction units (TU) per ml (82). The Swedish mutation enhances APP processing by the β-secretase BACE1, but the resulting APP-CTF is a wild-type substrate for γ-secretase (83). For the adenoviral transduction of NSCs, 1.5 × 10^6^ cells per well were seeded on laminin-coated 6-well plates in an NSC medium supplemented with EGF and FGF-2. The following day, cells were infected with one TU of adenovirus per cell for 3 h. 24 h after the infection, 1 ml of NSC medium containing EGF and FGF-2 and 10 μM phosphoramidon (Merck) was added and conditioned for another 24 h. As a control condition, cells were treated with the γ-secretase inhibitor LY-411575 (0.5 μM, Merck) to suppress Aβ generation. Aβ40 and Aβ42 peptide levels in conditioned media were quantified using the Meso Scale Diagnostics (MSD) electrochemical luminescence technology as previously described (84). Aβ peptides were captured with biotinylated mAb 4G8 against the Aβ mid-region on avidin-coated MSD plates, followed by detection with ruthenylated mAbs G2-10 or A387 specific for the C-termini of Aβ40 and Aβ42, respectively.

### Western blotting and immunocytochemistry

Cultured ES cells were lysed in Nonidet P40 buffer (50 mM Tris-HCl pH 7.8, 150 mM NaCl, 1% NP40 (v/v), with 1× EDTA-free complete protease inhibitor cocktail (Merck)). To detect γ-secretase subunits in the NSC lines, crude membrane extracts were prepared as previously described (85). In brief, the cells were collected and resuspended in 1 ml of hypotonic buffer (10 mM Tris, pH 7.4, 1 mM EDTA, 1 mM EGTA) containing 1× protease inhibitor cocktail and homogenized by passing five times through a 27-gauge needle (BD Microlance). To prepare a postnuclear supernatant, the homogenate was centrifuged at 1,000*g* for 15 min at 4 °C. The membranes were then isolated from the supernatant by centrifugation at 30,000*g* for 45 min at 4 °C. The membranes were resuspended in RIPA buffer (50 mM Tris-HCl pH 7.8, 150 mM NaCl, 1 mM EDTA, 1% Triton X-100 (v/v), 1% sodium deoxycholate (w/v), 0.1% SDS (w/v)) and cleared by a spin at 16,000*g* for 10 min at 4 °C. Total protein concentrations were determined with a bicinchoninic acid (BCA) protein assay kit (Pierce). Equal amounts of protein were separated by 12% or 4 to 12% Bis-Tris SDS-PAGE (ThermoFisher Scientific) and transferred onto PVDF membranes (Merck) by electroblotting. The membranes were blocked with 5% non-fat dry milk (w/v) in TBST (25 mM Tris, 137 mM NaCl, 2.7 mM KCl, 0.01% Tween-20 (v/v), pH 7.4) for 1 h at RT, and then incubated overnight at 4 °C with the primary antibody diluted in TBST + 0.02% NaN_3_ (w/v). Subsequently, a secondary antibody labeled with a near-infrared fluorescent dye (IRDye 800CW goat anti-mouse IgG or goat anti-rabbit IgG, LI-COR Biosciences) diluted in TBST containing 5% non-fat dry milk was added and incubated for 1 h at RT. Fluorescence signals were detected with the Odyssey CLx Imaging System and quantified using Image Studio Software 2.1 (LI-COR Biosciences).

For immunocytochemistry, NSCs derived from ES cell lines were seeded on laminin-coated glass coverslips. The cells were fixed for 15 min with 4% PFA (w/v) in PBS−/−, permeabilized for 10 min with 0.1 M glycine and 0.25% Triton X-100 (v/v) in PBS−/−, and blocked for 60 min with 30% goat serum (v/v, Merck) in antibody buffer (2% BSA (w/v), 5% sucrose (w/v) in PBS−/−), all steps at RT. Cells were incubated with the primary antibodies diluted in antibody buffer overnight at 4 °C. Subsequently, the following fluorophore-tagged secondary antibodies diluted in antibody buffer were applied in the dark for 1 h at RT: Alexa Fluor 488 goat anti-mouse (1:1,000, ThermoFisher Scientific Cat. No. A11001), and Cy3 goat anti-rat (1:1,000, Merck Cat. No. AP183C). After incubation with PBS−/− containing 0.1% Tween (v/v) and 0.1 μg DAPI (ThermoFisher Scientific) for 10 min, the coverslips were mounted on glass slides with FluorSave Reagent (Merck), cured for 24 h at RT in the dark, and analyzed with an Axio Imager ApoTome.2 microscope (Zeiss).

### Preparation of detergent-solubilized membrane fractions and co-immunoprecipitations

To prepare detergent-solubilized membrane fractions, confluent ES cells cultured on 10 × 10 cm dishes were washed and harvested in PBS−/−. The cells were collected by centrifugation at 3,000*g*, 3 min, 4 °C, resuspended in 10 ml homogenization buffer (50 mM HEPES, 250 mM sucrose, 5 mM EDTA, pH 7.0 containing 1× protease inhibitor cocktail), and disrupted using a nitrogen cavitation cell disruption vessel (Parr Instrument). Cell debris and nuclei were removed by centrifugation at 3,000*g*, 10 min, 4 °C, and cellular membranes were pelleted by centrifugation at 170,000*g*, 1 h, 4 °C. The pellet was washed once with 2 ml of ice-cold 0.1 M sodium bicarbonate (pH 11.4) and centrifuged again at 170,000*g*, 1 h, 4 °C. The membrane pellet was solubilized with 600 μl of 1% CHAPSO (w/v) in homogenization buffer for 90 min on a rotary wheel at 4 °C, and the solution was cleared by centrifugation at 170,000*g*, 1 h, 4 °C. The protein concentration in the supernatant was determined with a BCA assay kit and the solubilized membrane fractions were aliquoted and stored at −80 °C. For co-immunoprecipitation analysis, membrane fractions (160 μg total protein) were incubated with 80 μl Protein A/G PLUS-Agarose beads (Santa Cruz Biotechnology) and either anti-FLAG or anti-PSEN1-NTF antibodies overnight at 4 °C on a rotary wheel. The agarose beads were washed twice with 200 μl of homogenization buffer containing 0.25% CHAPSO, resuspended in 80 μl 1× SDS sample buffer, and incubated for 5 min at 65 °C. After centrifugation at 15,000*g*, 10 min, RT, the supernatant was analyzed by Western blotting as described above.

### γ-Secretase *in vitro* activity assays

γ-Secretase *in vitro* activity assays were performed as described previously with modifications (17). To generate a recombinant APP substrate, a DNA fragment encoding the C-terminal 99 amino acids of human APP with an additional Met at the N-terminus was generated by PCR and subcloned into the pET-DEST42 prokaryotic expression vector (ThermoFisher Scientific) in frame with C-terminal V5 and 6xHis tags. After induction with isopropyl β-D-1-thiogalactopyranoside, APP-C100-V5-6xHis was expressed in *E. coli* C43(DE3) cells (Merck) overnight at 18 °C and solubilized by 6 M guanidine hydrochloride in phosphate buffer (20 mM Na_2_HPO_4_, 500 mM NaCl, pH 7.2, containing 1× protease inhibitor cocktail). The APP substrate was purified via immobilized metal ion affinity chromatography using a 1 ml HiTrap TALON crude one column (Cytiva) and an AKTAprime plus chromatography system (Cytiva). The recombinant APP-C100-V5-6xHis was stored at −80 °C until use. As a source of γ-secretase activity, detergent-solubilized membrane fractions were prepared as described above. Membrane fractions (40 μg total protein) were incubated with Protein A/G PLUS-Agarose beads and either anti-FLAG, anti-PSEN1-NTF or anti-Nicastrin antibodies in 100 μl total volume of homogenization buffer containing 1% CHAPSO overnight at 4 °C on a rotary wheel. The agarose beads were washed twice with homogenization buffer containing 0.25% CHAPSO, resuspended in 30 μl of homogenization buffer containing 0.25% CHAPSO, 0.1% phosphatidylcholine (w/v), and 3 μM of APP-C100-V5-6xHis substrate, and incubated for 20 h at 37 °C. As a control condition, the γ-secretase inhibitor DAPT (1 μM, Merck) was included in the reaction mixture. The reaction was stopped by adding 10 μl of 4× SDS buffer and 5 min incubation at 65 °C. The beads were removed by centrifugation at 15,000*g*, 10 min, RT, and the supernatant was analyzed by Western blotting with the anti-V5 tag antibody.

### Statistical analysis

Statistical analysis was performed using Prism 9 (GraphPad Software), and data are represented as mean ± SD. Comparisons between three or more groups were analyzed by one-way ANOVA followed by Tukey’s post hoc test.

## Data availability

All data generated and analyzed during this study are included in the article and the supporting information.

## Supporting information

This article contains supporting information.

## Conflict of interest

The authors declare the following financial interests/personal relationships which may be considered as potential competing interests: D. B. and S. O. are employees of Asceneuron SA and own shares and stock options of the company. The authors declare that they have no conflicts of interest with the contents of this article.
