# Supplementary material for: Pathogenic Aβ production by heterozygous PSEN1 mutations is intrinsic to the mutant protein and not mediated by conformational hindrance of wild-type PSEN1
Source: J Biol Chem. 2023 Jun 30;299(8):104997. doi: 10.1016/j.jbc.2023.104997 (PMC10413157; doi:10.1016/j.jbc.2023.104997)
Supplement: Figs. S1–S3 and Tables S1–S4 [file mmc1.pdf]

Supporting information

Pathogenic Aβ production by heterozygous PSEN1 mutations is intrinsic to the mutant protein and not mediated by conformational hindrance of wild type PSEN1

Vanessa Kurth[1], Isabella Ogorek[1], Carolina Münch[1], Javier Lopez-Rios[3], Solenne Ousson[2], Sandra Lehmann[1], Katja Nieweg[4], Anton J.M. Roebroek[5], Claus U. Pietrzik[6], Dirk Beher[2], Sascha Weggen[1]

From the [1]Department of Neuropathology, Heinrich Heine University, Düsseldorf, Germany; [2]Asceneuron SA, Lausanne, Switzerland; [3]Centro Andaluz de Biología del Desarrollo (CABD), CSIC-Universidad Pablo de Olavide-Junta de Andalucia, Sevilla, Spain; [4]Institute of Pharmacology and Clinical Pharmacy, Philipps-University, Marburg, Germany; [5]Department of Human Genetics, KU Leuven, Leuven, Belgium; [6]Institute of Pathobiochemistry, University Medical Center of the Johannes Gutenberg-University, Mainz, Germany

Figure S1

Figure S2

Figure S3

Table S1

Table S2

Table S3

Table S4

Figure S1

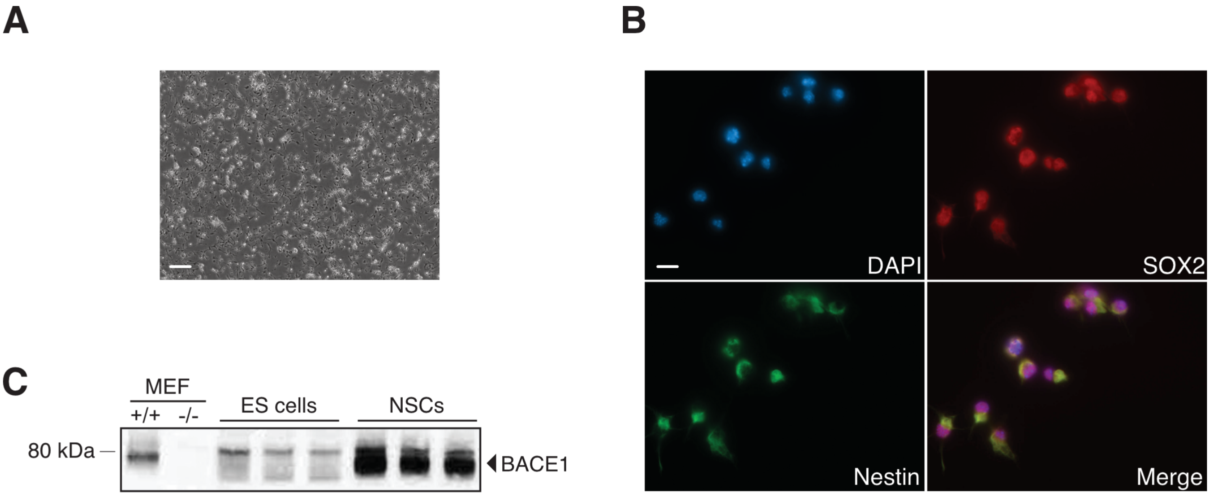

Figure S1. Neural stem cells (NSCs) derived from murine embryonic stem (ES) cells express NSC markers and upregulate BACE1 protein levels. *A*, ES cells engineered by dual recombinase-mediated cassette exchange (dRMCE) to harbor two wild type PSEN1 alleles were differentiated into NSCs. The cells displayed the typical morphology of NSCs growing in monolayer culture. Scale bar: 100 μm. *B*, Immunocytochemistry confirmed the uniform expression of the self-renewal transcription factor Sox2 and of the intermediate filament Nestin in the NSC cell population. Scale bar: 10 μm. *C*, Western blotting showed substantial upregulation of the β-secretase BACE1 in the NSCs compared to the parental ES cells. Murine embryonic fibroblasts derived from BACE1 knockout and wild type control animals (a gift from Bart de Strooper, KU Leuven, Belgium) were used to validate the specificity of the anti-BACE1 antibody (first two lanes).

Figure S2

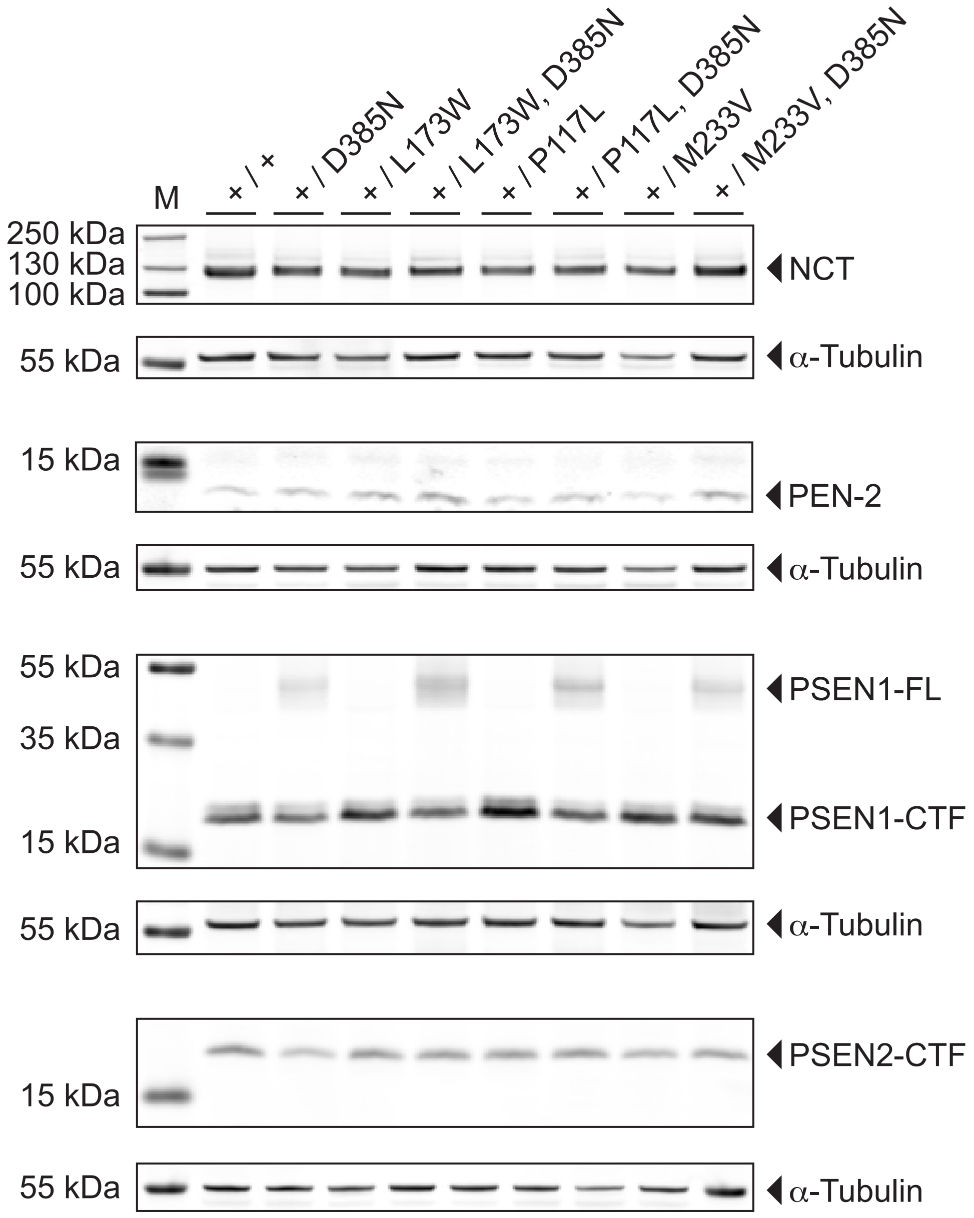

Figure S2. Protein levels of γ-secretase subunits in neural stem cell lines (NSCs) harboring heterozygous PSEN1 mutations. Crude membrane extracts were prepared from the different NSC lines and analyzed by Western blotting. The levels of the γ-secretase subunits NCT and PEN-2 were comparable in all NSC lines. The cell lines with only one catalytically active PSEN1 allele displayed lower PSEN1-CTF levels, which was accompanied by detectable levels of full-length PSEN1. Importantly, these NSC lines did not show compensatory upregulation of PSEN2 protein levels. Two independent biological experiments were performed (n = 2), and one representative experiment is shown.

Figure S3

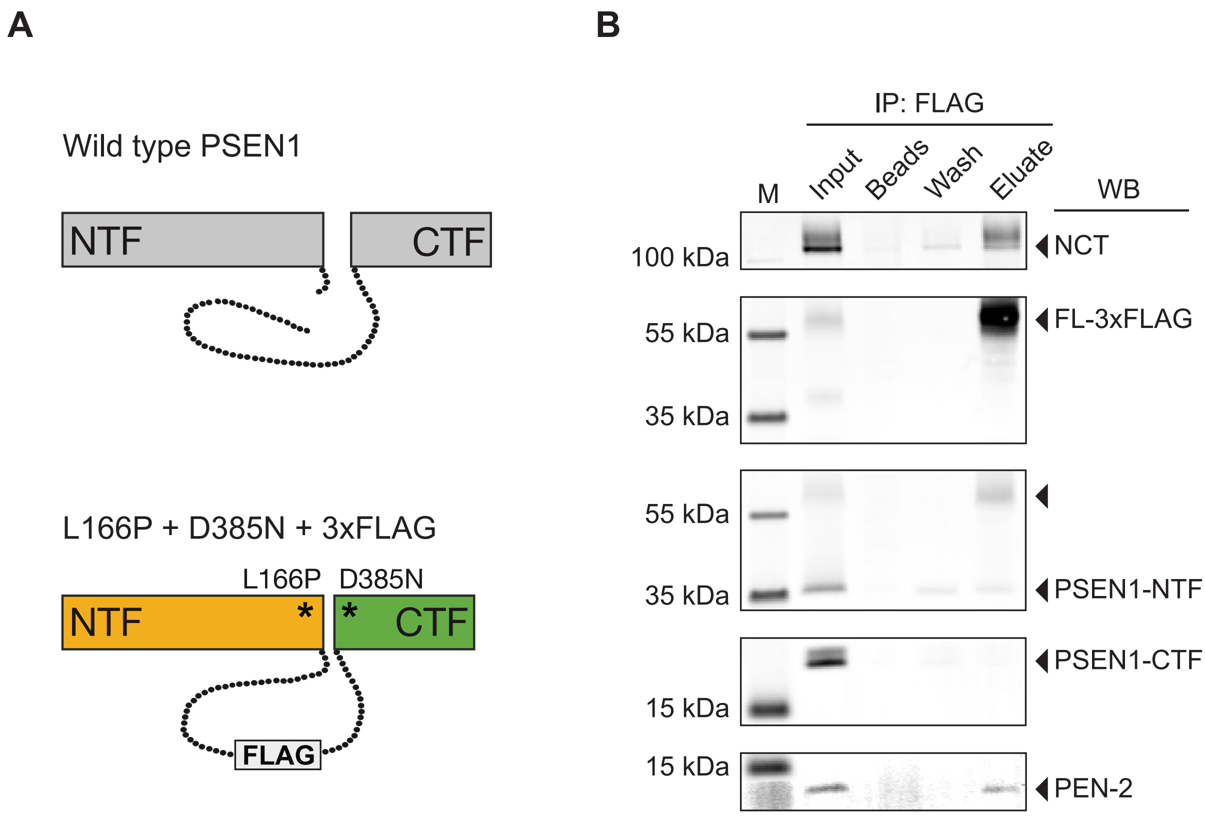

Figure S3. Lack of interaction between wild type and mutant PSEN1 containing the L166P mutation. *A*, dRMCE was used to generate an ES cell line harboring a wild type PSEN1 allele and a mutant allele with the eFAD-associated L166P mutation, the D385N mutation targeting the catalytic aspartate, and a 3xFLAG tag inserted into the cytosolic loop domain, permitting discrimination between the mutant and the wild type PSEN1 proteins by immunoprecipitation and Western blotting. *B*, CHAPSO-solubilized membranes were prepared and immunoprecipitations were performed with anti-FLAG antibody. Subsequently, the immunoprecipitated material (eluate) was analyzed by Western blotting with antibodies against the different γ-secretase subunits. Co-immunoprecipitation of the γ-secretase subunits Nicastrin (NCT) and presenilin enhancer 2 (PEN-2) was observed indicating that the eFAD mutant full-length protein (FL-3xFLAG), which migrated with a molecular weight slightly above 55 kDa, was normally incorporated into the γ-secretase complex. In contrast, co-immunoprecipitation of the PSEN1-NTF or the PSEN1-CTF expressed from the wild type allele was not detected. In control experiments, detergent-solubilized membranes were incubated with agarose beads but without the primary antibody (beads). In all experiments, non-specific binding of minimal amounts of the PSEN1-NTF to the bead material was observed. In the input lane, one-eighth of the total protein amount used for the immunoprecipitations was loaded. In the wash lane, one-fortieth of the total wash fraction (5 μl of 200 μl) was loaded. Three independent biological experiments were performed (n = 3), and one representative experiment is shown.

Table S1

Mutagenesis primers for the construction of pDREV-PSEN1 replacement vectors		
Primer	Genomic location	Sequence
PSEN1_P117L_For	Exon 5	CCT CTG TAG AAT CTA CAC CCT GTT CAC AGA AGA CAC TGA GAC
PSEN1_P117L_Rev		GTC TCA GTG TCT TCT GTG AAC AGG GTG TAG ATT CTA CAG AGG
PSEN1_L166P_For	Exon 6	GGT CAT CCA CGC CTG GCC TAT TAT TTC ATC TCT GTT
PSEN1_L166P_Rev		AAC AGA GAT GAA ATA ATA GGC CAG GCG TGG ATG ACC
PSEN1_L173W_For	Exon 6	TGG CTT ATT ATT TCA TCT CTG TTG TGG CTG TTC TTT TTT TCG TTC AT
PSEN1_L173W_Rev		ATG AAC GAA AAA AAG AAC AGC CAC AAC AGA GAT GAA ATA ATA AGC CA
PSEN1_M233V_For	Exon 7	TTA TGA TCA GTG CCC TCG TGG CCC TGG TAT TTA TC
PSEN1_M233V_Rev		GAT AAA TAC CAG GGC CAC GAG GGC ACT GAT CAT AA
PSEN1_D385N_For	Exon 11	GGA GTA AAA CTT GGA CTG GGA AAT TTC ATT TTC TAC AGT GTT C
PSEN1_D385N_Rev		GAA CAC TGT AGA AAA TGA AAT TTC CCA GTC CAA GTT TTA CTC C
PSEN1_3xFlag_For	Exon 10	GAC TAC AAA GAC CAT GAC GGT GAT TAT AAA GAT CAT GAC ATC GAT TAC AAG GAT GAC GAT GAC AAG CGC TCC ACT CCC GAG TCA AG
PSEN1_3xFlag_Rev		CTT GTC ATC GTC ATC CTT GTA ATC GAT GTC ATG ATC TTT ATA ATC ACC GTC ATG GTC TTT GTA GTC ATG AGG CCC CAG GTG ACT GT

Table S2

Validation primers for PSEN1 dRMCE		
Primer	Genomic locus specificity	Sequence
CDR-for	Conditional + Deleted + Replaced	TGG AGA AAA CCT TGG GTG AG
CD-rev	Conditional + Deleted	GCC GCT TGT CCT CTT TGT TA
CD-for	Conditional + Deleted	AGC AGA GCG GGT AAA CTG GC
CDR-rev	Conditional + Deleted + Replaced	TGT TTT CCA TGT ATG TCT TCT GG
C-for	Conditional	CCA ACC TGC CAT CAC GAG ATT
R-rev	Replaced	CTT GTA GCA CCT GTA TTT AT
R-for	Replaced	GCA ACC TCC CCT TCT ACG AG
Primer	Recombinase	Sequence
iCre-for	iCre coding sequence	GAC TAC CTC CTG TAC CTG CAA GCC AG
iCre-rev	iCre coding sequence	CTG CCA ATG TGG ATC AGC ATT CTC
FLPo-for	FLPo coding sequence	CAG CCT GAG CTT CGA CAT CGT GAA C
FLPo-rev	FLPo coding sequence	CTC AGG AAC TCG TCC AGG TAC ACC

Primer pair	Conditional locus	Deleted locus	Replaced locus	Wild type allele
CDR-for / CD-rev	**1448 bp**	**1448 bp**	no PCR product	no PCR product
CD-for / CDR-rev	**3585 bp**	**853 bp**	no PCR product	no PCR product
C-for / CDR-rev	**1460 bp**	no PCR product	no PCR product	no PCR product
CDR-for / R-rev	no PCR product	no PCR product	**1109 bp**	**994 bp**
R-for / CDR-rev	no PCR product	no PCR product	**1202 bp**	no PCR product

Table S3

PSEN1 dRMCE efficacy			
ES cell line	Total number clones	Positive clones	[%] Positive
PSEN1 wt	14	8	57
PSEN1 P117L	6	2	33
PSEN1 P117L + D385N	6	3	50
PSEN1 L166P + D385N + 3xFlag	6	3	50
PSEN1 L173W	6	3	50
PSEN1 L173W + D385N	6	4	67
PSEN1 M233V	6	3	50
PSEN1 M233V + D385N	6	2	33
PSEN1 M233V + D385N + 3xFlag	6	4	67
PSEN1 D385N	6	3	50
Σ	**68**	**35**	**52**

Table S4

Aβ levels in conditioned cell culture media		
Neural stem cell line	Aβ40 (pg/ml ± SD)	Aβ42 (pg/ml ± SD)
PSEN1 + / +	2247 ± 334	924 ± 412
PSEN1 + / D385N	2338 ± 421	804 ± 41
PSEN1 + / L173W	2162 ± 431	2499 ±666
PSEN1 + / L173W, D385N	2064 ± 405	735 ± 151
PSEN1 + / P117L	2160 ± 409	3056 ± 242
PSEN1 + / P117L, D385N	2115 ± 375	417 ± 144
PSEN1 + / M233V	2468 ± 495	1629 ± 239
PSEN1 + / M233V, D385N	2433 ± 421	588 ± 101

The means were calculated from 3-5 independent biological experiments (n = 3-5)
